# Levels of circulating myeloid subpopulations and of heme oxygenase-1 do not predict CD4^+^ T cell recovery after the initiation of antiretroviral therapy for HIV disease

**DOI:** 10.1186/1742-6405-11-27

**Published:** 2014-08-05

**Authors:** Lillian Seu, Gabriel M Ortiz, Trevor D Burt, Steven G Deeks, Jeffrey N Martin, Joseph M McCune

**Affiliations:** 1 Division of Experimental Medicine, Department of Medicine, University of California, San Francisco, CA 94110, USA; 2 Department of Bioengineering and Therapeutic Sciences, University of California, San Francisco, CA 94110, USA; 3 Division of Hospital Medicine, Department of Medicine, University of California, San Francisco, CA 94110, USA; 4 Division of Neonatology, Department of Pediatrics, University of California, San Francisco, CA 94110, USA; 5 Eli and Edythe Broad Center of Regeneration Medicine and Stem Cell Research, University of California, San Francisco, CA 94110, USA; 6 Positive Health Program, Department of Medicine, University of California, San Francisco, CA 94110, USA; 7 Department of Epidemiology and Biostatistics, University of California, San Francisco, CA 94110, USA

**Keywords:** HIV, HO-1, Immune activation, Monocytes, CD4^+^ T cell recovery

## Abstract

The level (or frequency) of circulating monocyte subpopulations such as classical (CD14^hi^CD16^-^) and non-classical (CD14^dim^CD16^+^) monocytes varies during the course of HIV disease progression and antiretroviral therapy (ART). We hypothesized that such variation and/or differences in the degree to which these cells expressed the immunoregulatory enzyme, heme oxygenase-1 (HO-1), would be associated with CD4^+^ T cell recovery after the initiation of ART. This hypothesis was tested in a cross-sectional study of four groups of HIV-infected subjects, including those who were seronegative, untreated virologic controllers [detectable viral load (VL) of <1000 copies/mL], untreated virologic non-controllers [VL > 10,000 copies/mL], and ART-mediated virologic controllers [VL < 75 copies/mL]. A longitudinal analysis of ART-treated subjects was also performed along with regression analysis to determine which biomarkers were associated with and/or predictive of CD4^+^ T cell recovery. Suppressive ART was associated with increased levels of classical monocyte subpopulations (CD14^hi^CD16^-^) and decreased levels of non-classical monocyte populations (CD14^dim^CD16^+^). Among peripheral blood mononuclear cells (PBMCs), HO-1 was found to be most highly up-regulated in CD14^+^ monocytes after *ex vivo* stimulation. Neither the levels of monocyte subpopulations nor of HO-1 expression in CD14^+^ monocytes were significantly associated with the degree of CD4^+^ T cell recovery. Monocyte subpopulations and HO-1 gene expression were, however, restored to normal levels by suppressive ART. These results suggest that the level of circulating monocyte subpopulations and their expression of HO-1 have no evident relationship to CD4^+^ T cell recovery after the initiation of ART.

## Background

Circulating myeloid cells are a heterogeneous population of monocytes and dendritic cells with diverse immunoregulatory capacities [1]. CD14^dim^CD16^+^ "non-classical" monocytes have gene expression profiles that are generally considered to be pro-inflammatory in nature and are found at higher frequencies in the blood of subjects with chronic inflammatory diseases such as HIV infection [2]. CD14^hi^CD16^-^ "classical" monocytes comprise the bulk of the circulating myeloid pool and are anti-inflammatory in function, as demonstrated by their low potential to migrate to inflammatory sites [3], their production of IL-10 after stimulation with lipopolysaccharide (LPS) [4], and their propensity to mature into tolerogenic antigen presenting cells (APCs) in the context of appropriate cytokine stimulation [5]. Consistent with their pro-inflammatory properties, non-classical CD14^dim^CD16^+^ monocytes are found at 2- to 8-fold higher levels in HIV-infected subjects compared to uninfected controls [6]. Additionally, untreated HIV-infected subjects with elevated frequencies of circulating CD14^hi^CD16^+^ "intermediate" monocytes have increased viral loads and decreased CD4^+^ T cell counts which decline after virologically suppressive ART [7]. How these changes in monocyte subpopulations might impact SIV/HIV disease progression remains unclear.

Heme oxygenase-1 (HO-1), the rate-limiting enzyme in the catabolism of heme, has potent immunoregulatory activity. HO-1 activity within myeloid cells is generally thought to be anti-inflammatory- resulting in decreased TNFα expression, increased IL-10 expression, and is associated with differentiation into tolerogenic APCs [8]. We have previously demonstrated that monocytes represent one of the most prevalent sources of HO-1, and HO-1 inhibitors cause monocyte-dependent T cell proliferation *in vitro*[9]. HO-1 levels are elevated in the peripheral blood cells of HIV-infected individuals [10], and functional HO-1 promoter polymorphisms correlate with circulating markers of inflammation in ART-treated subjects [11]. Given these data, we wondered whether increased levels of certain monocyte subpopulations and/or of their expression of HO-1 might be generally anti-inflammatory in nature and thereby facilitate the CD4^+^ T cell response to suppressive ART. Altering the balance of the immune system towards a less activated state (e.g., by increasing the frequency of the anti-inflammatory classical monocytes) may prevent immunopathology associated with chronic HIV disease and, ultimately, improve HAART-mediated CD4^+^ T cell recovery. In this study, we sought to determine if CD4^+^ T cell recovery after the initiation of ART was predicted by the frequency of monocyte subpopulations and/or of HO-1 expression in CD14^+^ monocytes.

## Results

### Antiretroviral therapy is associated with an increased frequency of classical monocytes

**Figure 1:**
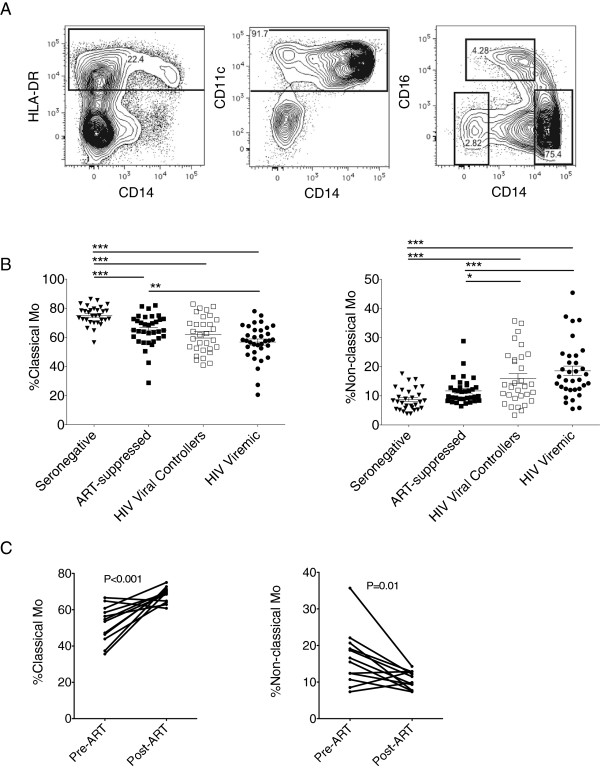
**Prolonged ART results in an increased frequency of classical monocytes. (A)** Thawed PBMCs from HIV patients were stained with antibodies recognizing cell-surface and intracellular myeloid markers. Analysis was performed by sequentially gating on live cells, singlets (FSC-A/FSC-H), non-lymphocyte (SSC-A high/FSC-A high), lineage negative (CD3^-^ CD19^-^ CD56^-^), and HLA-DR and CD11c positive populations. Myeloid cells were further defined by expression of CD14 and CD16 into three subsets (CD14^hi^CD16^-^, CD14^dim^CD16^+^, and CD14^-^CD16^-^ cells). Frequencies of CD14^hi^CD16^-^ classical monocytes (lower right gate), CD14^dim^CD16^+^ non-classical monocytes (upper left gate), and CD11c^+^ CD14^-^CD16^-^ mDCs (lower left gate) and in relation to the parent myeloid gate were calculated. **(B)** Column statistics were performed by 1-way ANOVA on patients described in Table 1. Statistical significance is denoted as *p < 0.05, **p < 0.01, and ***p < 0.001. **(C)** Myeloid subpopulations were measured during pre-ART to post-ART time points from thawed PBMCs of ART-treated subjects (see "pre- ART-suppressed" subjects described in Table 2). Student’s paired t-test was performed and corresponding p values are described.

As shown in Figure 1A, three blood myeloid subpopulations were defined by expression of HLA-DR, CD14, CD16, and CD11c: CD14^hi^CD16^-^ classical monocytes, CD14^dim^CD16^+^ non-classical monocytes, and CD11c^+^ myeloid dendritic cells (mDCs) (Figure 1A, and Additional file 1: Figure S1A) [12].

To determine the relative frequency of various myeloid subpopulations in the context of HIV disease, PBMCs from untreated viral controllers, untreated viremics, ART-suppressed subjects, and HIV-seronegative subjects (Table 1) were studied by flow cytometry. The highest circulating frequencies of classical monocytes were observed in HIV-seronegative subjects, a group known to have low levels of T cell activation, while the highest frequencies of pro-inflammatory non-classical monocytes were observed in untreated viremic subjects, a group known to have high levels of T cell activation (Figure 1B and Additional file 2: Figure S2) [13]. These results confirm and extend existing reports of altered monocyte populations during HIV disease progression [7,14].

**Table 1:** Clinical descriptions for cross-sectional HIV patients

**Patients**	***N***	**VL (copies/mL)**^#^	**CD4 (cells/ul)**^#^	**Mean age**^#^	**Male%**
HIV Viral Controllers^a^	31	75 (75–184.3)	710 (514–945.5)	45.5 (43–52.7)	64.5%
HIV Viremic^b^	34	89246 (45295.5–174381)	304 (202.2–451)	43 (39.2–47.7)	79.4%
ART-suppressed^c^	34	75 (50–75)	388.5 (313.5–500.2)	47 (41.7–50)	79.4%
Seronegative	30	NA	NA	42 (37.2–45.7)	100.0%

^a^HIV viral controllers were defined as untreated subjects with at least three plasma HIV VL < 1,000 copies/mL during a 12 month period.

^b^HIV viremics were defined as untreated individuals with VL > 10,000 copies/ml.

^c^ART-suppressed subjects had VL < 75 copies/mL for a period between 2–4 years after treatment initiation, with only one viral blip >1000 copies/mL permissible during the analysis time.

^#^Values are expressed as median ± interquartile range.

To understand the cellular characteristics of circulating monocyte populations in more detail, we performed a comprehensive analysis of the geometric mean fluorescence intensities (gMFI) of various myeloid markers in these defined monocyte populations within patients on early ART n = 24 (Early ART suppressed, Table 2). The following markers were assessed: HLA-DR (a Class II major histocompatibility receptor [15]), CD11c (a type 1 transmembrane integrin), CD11b (a Mac-1 integrin forming the complement receptor 3), and CD33 (a myeloid-restricted transmembrane protein of the sialic acid binding Ig-like lectin family). (Additional file 3: Figure S3) The CD14^hi^ classical monocytes had the highest levels of CD11b and CD33 expression (P < 0.0001) whereas the CD14^dim^CD16^+^ non-classical monocytes had the highest levels of CD11c expression (P < 0.0001). CD11c^+^ mDCs that were CD14^-^CD16^-^ did not display high levels of CD11b, CD11c, or CD33 (Additional file 3: Figure S3). These findings indicate that classical and non-classical monocytes can be further characterized by cell surface expression levels of CD11c, CD11b, and CD33, molecules that have implications on trafficking through the circulation and in tissue sites of infection [16]. A recent study showed that other monocyte markers such as CD62L, CD115, neopterin, and soluble CD163 and CXCL10 are dynamically altered in HIV patients [17].

**Table 2:** **Biomarkers in pre-ART suppressed subjects prior to and during suppressive ART**^**a**^

	**n = 12**	**Pre-ART timepoint [Median (IQR)]**	**Post-ART timepoint [Median (IQR)]**	**P value**^**a**^
Gender		83.30%		/
Age	Years	46.5 (41.5–47)	49 (43.2–50)	/
Viral Load	copies/mL	33,123 (19,383–100,092)	75 (75–75)	0.03
CD4^+^ T cells	Total (cells/uL)	173.5 (149.3–251.8)	435 (343.8–594.3)	0.0006
CD8^+^ T cells	Total (cells/uL)	1320 (792–1630)	1561 (1105–2313)	ns
Activation (+%)	CD4^+^CD38^+^HLADR^+^	13.0 (9.6–26.6)	3.6 (2.6–4.7)	0.0007
	CD8^+^CD38^+^HLADR^+^	22.5 (13.9–24.1)	6.8 (5.2–10.2)	0.0007
SSC^++^Lin^-^ (+%)	Classical Monocytes (HLADR^+^CD11c^+^CD14^hi^CD16^-^)	54.1 (44.5–60.2)	69.2 (64.3–71.1)	0.0009
	Non-Classical Monocytes (HLADR^+^CD11c^+^CD14^dim^CD16^+^)	16.0 (11.1–20.3)	10.7 (8.1–12.9)	0.01
PBMC gene transcript expression	HO-1/HPRT	142.5 (98.0–182)	78.8 (56.5–104.5)	0.05

^a^Pre-ART suppressed subjects were un-treated subjects with un-suppressed viral load.

ART is known to result in both decreases in plasma virus as well as in T cell activation levels [13]. Based on our cross-sectional analysis (Figure 1B), classical monocytes were hypothesized to increase over time on ART. To directly test this hypothesis, myeloid cell subpopulations were analyzed longitudinally over time in the "pre-ART" subjects (described in Table 2). Pre-ART subjects (n = 12) were not on ART and had a median viral load of 33,123 copies/mL (IQR 19,383 - 100,092) and a median CD4 count of 173.5 cells/mm3 (149.3 - 251.8). After 2.5 years on suppressive therapy (IQR 1.7 – 3), viral loads were at levels below 75 copies/mL, the limit of viremia detection. During this time, classical monocyte frequencies increased from 54.1% (IQR 44.5-60.2%) to 69.2% (IQR 64.3 – 71.1%) (P < 0.001) whereas non-classical monocyte frequencies decreased from 16.0% (IQR 11.1- 20.3%) to 10.7% (IQR 8.1 – 12.9%) (P = 0.01) (Figure 1C). These results corroborate previous findings where non-classical monocyte populations appear to diminish after suppressive ART [7,18].

### HO-1 is induced in CD14^+^ monocytes by cobalt protoporphyrin IX (CoPP)

**Figure 2:**
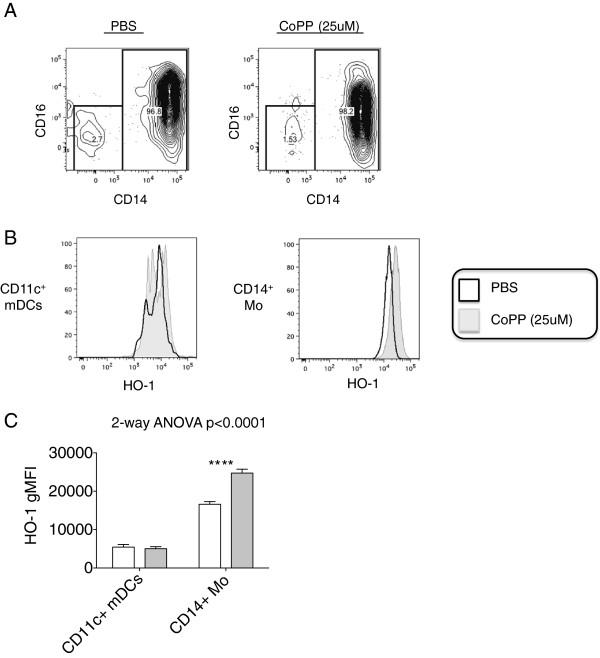
**CD14**^**+ **^**monocytes are the main site of HO-1 induction after *ex vivo *CoPP stimulation.** Thawed PBMC samples from Early ART Patients (Table 3) (n = 24) were analyzed for cell surface expression of monocyte markers. **(A)** Scatter plots showing the expression of CD14 and CD16 after monocyte cell culture in PBS (left) and stimulation with CoPP (right) on UpCell™ plates are depicted **(B)** Histograms representing the fluorescence intensities of HO-1, CD14, CD16, and CD11c in CD14^+^ monocytes and in CD11c^+^ mDCs after treatment with saline or CoPP are shown. Thawed PBMCs were cultured with saline or 25 μM CoPP for 48 hours on UpCell™ plates, harvested, and then stained with antibodies recognizing cell-surface and intracellular myeloid markers. Data from a representative subject are shown. **(C)** CoPP-induced changes in HO-1 among CD14^+^ monocytes or CD11c^+^ DCs. The ****symbol represents a p < 0.0001 for differences in culture conditions (saline vs. CoPP).

To determine which of the various myeloid subpopulations circulating in HIV-seropositive subjects might be the main producers of HO-1, stimulation experiments were carried out to detect intracellular HO-1 before and after *ex vivo* induction with Cobalt protoporphyrin IX (CoPP) [9]. At baseline prior to CoPP stimulation, HO-1 expression was higher in the CD14^+^ monocyte subpopulation than in the CD11c^+^ mDC population (Figure 2B).

Cell culture and stimulation resulted in classical and non-classical monocyte populations to combine into one subpopulation of CD14^+^ monocytes with intermediate expression of CD14 and CD16 compared to baseline expression patterns (Figure 2A). Upon CoPP stimulation, the CD14^+^ monocytes expressed higher HO-1 levels compared to their PBS (Figure 2C; p < 0.0001, 2-way ANOVA post-test). Consistent with prior findings [9,11], our observations suggest that CD14^+^ monocytes are the most biologically relevant producers of HO-1 in the peripheral blood.

### Antiretroviral therapy is associated with decreased PBMC levels of HO-1

**Figure 3:**
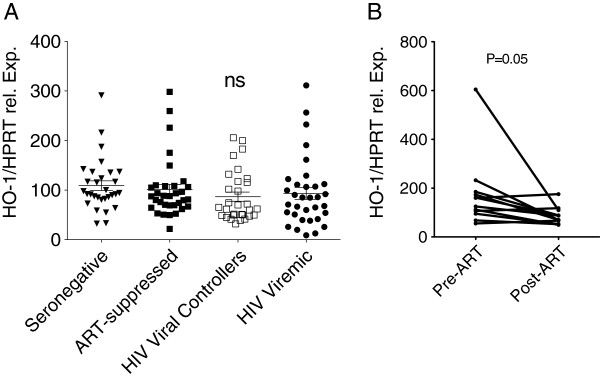
**Antiretroviral therapy is associated with decreased PBMC mRNA levels of *HMOX1*. (A)** Cross-sectional analysis of the relative transcript expression levels of *HMOX1/HPRT* in patients described in Table 1. Column statistics were performed by 1-way ANOVA. Statistical significance is denoted as *p < 0.05, **p < 0.01, and ***p < 0.001. **(B)** Longitudinal changes in relative transcript expression levels of HO-1/HPRT were measured during pre-ART to post-ART time points from thawed PBMCs of ART-treated subjects (see "pre-ART suppressed subjects" described in Table 2). Student’s paired t-test was performed with p value = 0.05.

Relative transcript levels of HO-1 were assessed from thawed PBMCs from untreated viral controllers, untreated viremic subjects, ART-treated subjects, and HIV-seronegative subjects (Table 1). There were no statistically significant differences in the levels of HO-1 transcript across all patient groups (Figure 3A).

We tested whether *HMOX1* transcript changed after the initiation of suppressive ART in patients that were followed longitudinally (Table 2). *HMOX1* relative gene expression levels decreased from 142.5 (98.0 - 182) to 78.8 (56.5 - 104.5) (P = 0.05) (Figure 3B).

### Circulating monocyte frequencies and levels of HO-1 expression are not predictive of better CD4^+^ T cell recovery in ART-treated subjects

**Figure 4:**
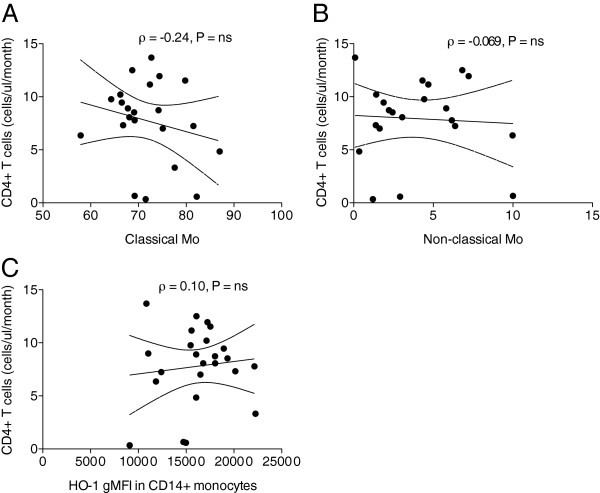
**Circulating monocyte frequencies measured at time points early after ART initiation are not predictive of better subsequent CD4**^**+ **^**T cell recovery in ART-treated subjects.** Thawed PBMC samples from Early ART Patients (Table 2) were analyzed for cell surface expression of monocyte markers. Regression analysis of CD4^+^ T cell recovery between time point 1 and time point 2 was performed using the Spearman rank correlation. Rho coefficient (ρ) reveals lack of association between CD4^+^ T cell recovery and the following parameters measured at ART time point 1: **(A)** percentage of classical monocytes, **(B)** percentage of non-classical monocytes, and **(C)** HO-1 gMFI levels in CD14^+^ monocytes.

CD4^+^ T cell recovery was longitudinally assessed in early ART-suppressed cohort (n = 24, Table 2). All subjects achieved durable ART-mediated viral suppression by seven months of treatment. We hypothesized that subjects with higher pro-inflammatory non-classical CD14^dim^CD16^+^ monocyte frequencies may experience lower CD4^+^ T cell gains during suppressive ART (Table 3). We performed regression analysis to determine if classical monocytes or non-classical monocytes measured at a time point early after ART initiation may be predictive of the subsequent rate of CD4^+^ T cell count gain (cells/mm3/month). There was no strong evidence of a significant association between either the level of classical monocytes (Spearman’s rho ρ = -0.24, P < ns) (Figure 4A) or of non-classical monocytes (Spearman’s rho ρ = -0.069, P < ns) and CD4^+^ T cell counts (cells/mm3) measured during early or late ART, nor with the subsequent rate of gain in CD4^+^ T cell count (cells/mm3/month) (Figure 4B).

**Table 3:** **Cellular expression levels (+ %) of immunological biomarkers in early- ART suppressed HIV-infected subjects**^**a**^

	**n = 24**	**ART time point 1**	**ART time point 2**	**P value**^**a**^
Gender		58.30%		/
Age	Years	46 (42–48.7)	48 (44–50)	/
Length of time on ART	Months	6.4 (4.8–13.9)	29.3 (27.3–38.9)	/
Viral Load	copies/mL	75 (56.2–75)	75 (56.2 - 75)	ns
CD4^+^ T cells	Total (cells/uL)	300.0 (222.5–360.7)	425 (328.25–513.5)	<0.0001
CD8^+^ T cells	Total (cells/uL)	1085 (710–1396)	1114 (750.3–1593)	ns
SSC^++^Lin^-^ (+%)	CD11c^+^ mDCs (HLADR^+^CD11c^+^)	3.9 (2.7–5.4)	2.8 (2.3–4.4)	ns
	Classical Monocytes (HLADR^+^CD11c^+^CD14^hi^CD16^-^)	70.3 (67.8–75.1)	78.8 (74.35–83.1)	0.004
	Non-Classical Monocytes (HLADR^+^CD11c^+^CD14^dim^CD16^+^)	3.0 (1.6–6.2)	3.8 (2.3–5.7)	ns
Classical Monocytes (HLADR^+^CD11c^+^CD14^hi^CD16^-^)	HLA-DR gMFI	14375(10569–16546)	12516 (10343–15220)	ns
	CD11b gMFI	31139 (26954–34508)	31096 (27432–34876)	ns
	CD11c gMFI	13514 (10406–14970)	12069 (10430–13956)	0.008
	CD33 gMFI	29624 (20462–36580)	29938 (19164–37687)	ns
Non-Classical Monocytes (HLADR^+^CD11c^+^CD14^dim^CD16^+^)	HLA-DR gMFI	12311 (10047–14272)	12993 (11328–14673)	ns
	CD11b gMFI	2123 (1726–3767)	2421 (1756–2957)	ns
	CD11c gMFI	21517 (17876–24957)	22780 (20620–24761)	ns
	CD33 gMFI	8678 (6510–11084)	8956 (6472–11433)	ns
CD14^+^ monocytes	HO-1 gMFI	16290 (14784–18027)	15785 (13875–18535)	ns

^a^Early HAART-suppressed subjects had VL <100 copies/mL after initial viral suppression, with only one blip of >1000 copies/mL permissible during the time frame of analysis on HAART. Early HAART time point refers to a period between 2–10 months of treatment initiation, at a time when VL had achieved an undetectable level.

To perform more in-depth analysis of monocyte populations and their role in CD4^+^ T cell recovery, we further analyzed HO-1 [9] and tested the possibility that its expression within CD14^+^ monocytes might be high in HIV subjects with improved CD4^+^ T cell recovery after ART in an analysis similar to a prior cohort study [19]. HO-1 expression in CD14^+^ monocytes measured at the first time point available after complete viral suppression (ART time point 1) was not significantly associated with more robust CD4^+^ T cell recovery over the course of ART (Figure 4C).

In summary, these results demonstrate that neither circulating levels of monocyte populations nor high HO-1 expression in classical monocytes measured at a time point early after ART initiation predict greater CD4^+^ T cell gains at later ART time points, despite being differentially altered during the course of untreated HIV disease.

## Discussion

Immune activation is a cardinal feature of HIV disease and contributes to pathologic outcomes before and after the initiation of ART [20]. In each instance, it is not clear if immune activation is due directly to viral replication and/or to the host response to such replication. It is clear, however, that the higher the level of activation, the faster the course of disease progression in untreated subjects [13,20] and the lower the level of CD4^+^ T cell recovery in those provided ART.

We demonstrate that suppressive ART restores homeostatic levels of monocyte population frequencies (Figure 1B, C). There may be multiple explanations for these findings. For example, diminished levels of CD14^+^ myeloid cells could arise if CD14 cell surface receptors are actively shed after activation. The CD14 molecule serves as the bacterial lipopolysaccharide (LPS) receptor that is cleaved when engaged by LPS, resulting in circulating soluble CD14. The increase in the frequency of classical monocytes observed after suppressive ART may reflect decreased plasma LPS, allowing persistent expression of CD14 on these cells [21]. Moreover, and as suggested by the current studies, expression of immunomodulatory enzymes such as HO-1 by classical monocytes may contribute to control of immune activation after ART-mediated viral suppression. Cellular phenotyping of monocytes was performed on cryopreserved samples, and previous studies have comparable results upon the freeze-thawing process of peripheral blood mononuclear cells [22,23].

We hypothesized that frequencies of circulating monocyte subpopulations may not only be differentially altered during the course of HIV disease [7] but may also predict the rate of CD4^+^ T cell recovery in patients that are suppressed on ART. Our study was designed such that the first time point of analysis occurred after the early months of ART (when there is substantial patient-to-patient variation in the kinetics of suppression of viremia [24] and of T cell redistribution [25]) as well as after the virus load has been effectively suppressed (thereby avoiding the confounding effects of unsuppressed and variable viral loads that would have otherwise been a major driver of most if not all of the measured parameters [13]). In the cohort studied, however, circulating classical or non-classical monocyte percentages were not predictive of CD4^+^ T cell recovery in early ART-suppressed patients (Figure 4A, B). However, the possibility of missing an association due to low statistical power could be related to the relatively small sample size and/or small effect size of CD4^+^ T cells (cells/uL) gain per patient.

A similar study looking at a panel of candidate T cell biomarkers within the same patient cohort described in Table 2 (Early ART suppressed) [19] demonstrated that poor levels of CD4^+^ T cell recovery are predicted by high levels of CD8^+^ T cells with a senescent phenotype, i.e., increased cell surface expression of CD57 and/or decreased cell surface expression of CD27 and of CD28.

The altered monocyte populations observed during the context of HIV disease have further implications as they may constitute a viral reservoir. It appears that CD16-positive monocytes (5% of monocyte population [2]) are both more susceptible to infection and preferentially harbor the virus long-term [26]*in vitro*. We have also reported that immunoregulatory enzymes like HO-1 and indoleamine 2, 3-dioxygenase (IDO), may have beneficial effects in HIV-seropositive subjects [27]. While HO-1 expression in CD14^+^ monocytes was not predictive of CD4^+^ T cell recovery when measured at time points early after ART-mediated viral suppression, (Figure 4C) suppressive ART did restore homeostatic levels of *HMOX1* gene expression (Figure 3B). HO-1 is an important immune modulator, with effects that are anti-proliferative, anti-oxidant, anti-apoptotic, and cytoprotective. (Reviewed in [28]) HO-1 deficiency results in macrophages that produce greater amounts of TNFα, IFNα, IL-6, and IL-2 after LPS stimulation in mouse models [29]. Reciprocally, increased levels of HO-1 are associated with decreased levels of HIV replication in monocytes *in vitro*[8]. Therefore, PBMC HO-1 levels may be reflective of the overall immune activation state, resulting in parallel decreases in both parameters over time on ART.

## Conclusions

We performed a comprehensive assessment of the relationship between circulating monocyte populations and HIV disease outcome among ART patients (as defined by CD4 reconstitution) in a cohort of well-characterized HIV- infected and -uninfected adults. We show that suppressive ART restores homeostatic levels of monocyte population frequencies as well as HO-1 gene expression levels. These results suggest that monocyte populations may be dysregulated during chronic untreated HIV disease, that suppressive ART restores their frequencies to normal levels, and that, at least at this level of discrimination, neither the levels of circulating monocyte populations, nor the levels of the immunoregulatory enzyme, HO-1, do not predict CD4^+^ T cell recovery after the initiation of ART but rather parallel the overall immune activation levels. This homeostatic recovery of different monocyte populations upon successful ART may contribute to the extent of immunological restoration and overall disease management in chronic HIV patients.

## Methods

### Human subjects

Healthy, HIV-seronegative adults (n = 30) and HIV-seropositive subjects (n = 135) were recruited from the San Francisco-based UCSF SCOPE (Study of the Consequences of the Protease Inhibitor Era) cohort. All subjects provided written informed consent for all biologic specimens and clinical data used in this study, and research records were kept confidential meeting specifications for this project approved by the UCSF Committee on Human Research (IRB #10-01330, reference #046371). For cross-sectional studies, HIV-infected subjects were categorized into four groups: virologic controllers (VL < 1000 copies/mL) (n = 31), virologic non-controllers (VL > 10,000 copies/mL) (n = 34), and ART-mediated virologically suppressed subjects (n = 34) (Table 1). Pre-ART subjects (n = 12) were followed longitudinally at time points from a time prior to ART initiation as well as 2.5 years post-suppressive therapy (Table 2). "Early ART" subjects (n = 24), were followed at a time point from early ART (median 6.4 months, interquartile range [IQR] 4.8 – 13.9 months) as well as 1 – 2 years of follow-up (median 19.8 months, IQR 18.3 – 24.6 months) were used for analysis as described in Table 3.

### Isolation of plasma and primary peripheral bloods cells

Peripheral blood was drawn into EDTA tubes and centrifuged, after which the plasma fraction was frozen at -80°C until use. Peripheral blood mononuclear cells (PBMCs) were isolated from peripheral blood drawn into sodium heparin tubes by density centrifugation using Histopaque®-1077 (Sigma Aldrich, Saint Louis, MO). PBMCs were washed with RPMI 1640 (Life Technologies, Rockville, MD) supplemented with 10% fetal bovine serum (FBS, Gemini Bio-Products, Woodland, CA), 1% penicillin/streptomycin (Mediatech, Washington, DC), and 2 mM L-glutamine (Mediatech) (hereafter referred to as R10 medium), frozen in small aliquots of FBS with 10% DMSO (Sigma), and stored in a liquid nitrogen cryofreezer until use.

### PBMC preparation

Cryopreserved PBMCs were rapidly thawed and washed in R10 medium. Viable PBMCs that excluded trypan blue (Sigma) were counted by direct microscopic visualization using a hemacytometer, pelleted, and then re-suspended at an adequate cell number for subsequent experiments.

### HO-1 induction experiments

Cobalt protoporphyrin IX (CoPP) was purchased as a powder from Frontier Scientific (Park City, Utah), dissolved in 0.1 mM NaOH (Sigma), and then titrated to a pH of 7.6. PBMCs were cultured on Upcell™ 96 F MicroWell plates (Nalge Nunc, Rochester, NY), either with saline or 25 μM CoPP in R10 at 37°C for 48 hours. After incubation, adherent cells were harvested by incubating the plates at 25°C for 20 minutes.

### Flow cytometry antibody labeling

The monoclonal antibodies (mAbs) used in this study were purchased from Abcam (Cambridge, MA), BD Biosciences (Franklin Lakes, NJ), Beckman Coulter (Indianapolis, IN), BioLegend (San Diego, CA), eBiosciences (San Diego, CA), and Invitrogen (Carlsbad, CA), and are summarized in detail in Additional file 4: Table S1. Briefly, cells were washed in staining buffer containing PBS with 2% FBS and 20 mM EDTA (Sigma) before a 30 minute incubation at 4°C in the presence of a cocktail of mAbs as well as Amine-Aqua Dead Cell Stain (Invitrogen). Afterwards, cells were washed with staining buffer. Intracellular staining with the HO-1 antibody (Abcam) alongside a secondary antibody (Invitrogen) was performed for one hour at 4°C in cells that were fixed and permeabilized in BD Cytofix/Cytoperm, according to the manufacturer’s protocol. All cells underwent a final wash prior to fixation in 1% paraformaldehyde (Sigma). Data were acquired on an LSR II flow cytometer (BD) and analyzed using FlowJo software (Treestar, Ashland, OR). Gates for flow cytometric analyses were based on "fluorescence-minus-one" control stains.

### Quantitative PCR

For quantitative PCR analysis, PBMCs were harvested after culturing for 48 hours and RNA was isolated using TRIzol® Reagent (Invitrogen, Carlsbad, CA). Total cellular RNA (0.2 μg) was used for cDNA synthesis using Oligo-dT primers and Reverse Transcriptase from Omniscript (Qiagen). Relative expression levels of *HMOX1* mRNA were measured by quantitative RT-PCR using validated Taqman® Gene Expression assay mixes for human *HMOX1* (Hs00157965_m1) and human *HPRT* (Hs99999909_m1) according to the manufacturer’s protocol (Applied Biosystems). An AB Step One Plus instrument was used for amplification and detection, and the 2-ΔΔCT calculation was used to measure *HMOX1* gene expression relative to *HPRT*[11].

### Statistical analyses

Linear regressions, correlation analyses, ANOVAs, and paired T tests were made across biomarkers of interest using GraphPad Prism v5.0d (Graphpad Software, La Jolla, CA, USA). For flow cytometric expression levels, geometric mean fluorescence intensities (gMFI) were used. CD4^+^ T cell immune reconstitution was measured by subtracting the value of each patient’s CD4^+^ T cell count (cells/uL) from the early (median 6.4 months) from the late (median 29.3 months) ART timepoint, then dividing by the total number of months spanning that time period.

## Competing interests

The authors report no conflict of interest for this study.

## Authors’ contributions

LS and GMO carried out the immunological studies, acquired and performed the data analyses, and drafted the manuscript. TDB participated in the design of the immunological studies, and provided technical expertise. SGD performed the clinical design of the study. JNM performed the clinical statistical analyses. JMM conceived of the study, and participated in its design and coordination and drafted the manuscript. All authors read and approved the final manuscript.

## Supplementary Material

Additional file 1: Figure S1 Three blood myeloid subpopulations were defined by expression of HLA-DR, CD14, CD16, and CD11c: CD14^hi^ CD16^-^ classical monocytes, CD14^dim^CD16^+^ non-classical monocytes, and CD11c^+^ myeloid dendritic cells (mDCs) (Flow plots from ART patients described in Table 3). Click here for file

Additional file 2: Figure S2 Antiretroviral therapy is associated with decreased T cell activation. Column statistics were performed by 1-way ANOVA on the % expression of HLA-DR and CD38 on (A) CD4^+^ T cells and (B) CD8^+^ T cells. Click here for file

Additional file 3: Figure S3 Monocyte populations are defined by distinct cell surface receptors. Thawed PBMC samples from Early ART Patients (ART time point 1 in Table 3) (n = 24) were analyzed for cell surface expression of monocyte markers. Plots depict staining intensity (geometric mean fluorescence intensity) of various myeloid markers (HO-1, HLA-DR, CD11b, CD11c, CD33, and CD124) in CD14^hi^CD16^-^, CD14^dim^CD16^+^, and CD14^-^CD16^-^ cells (column statistics performed by 1-way ANOVA, with asterisks representing the significance to the decimal place of the p value). Click here for file

Additional file 4: Table S1 Antibodies used for flow cytometry staining. Click here for file
