# Supplementary material for: Levels of circulating myeloid subpopulations and of heme oxygenase-1 do not predict CD4+ T cell recovery after the initiation of antiretroviral therapy for HIV disease
Source: AIDS Res Ther. 2014 Aug 5;11:27. doi: 10.1186/1742-6405-11-27 (PMC4150425; doi:10.1186/1742-6405-11-27)
Supplement: Additional file 1: Figure S1 — Three blood myeloid subpopulations were defined by expression of HLA-DR, CD14, CD16, and CD11c: CD14hi CD16- classical monocytes, CD14dimCD16+ non-classical monocytes, and CD11c+ myeloid dendritic cells (mDCs) (Flow plots from ART patients described in Table 3). [file 1742-6405-11-27-S1.pdf]

Supplemental Figure 1

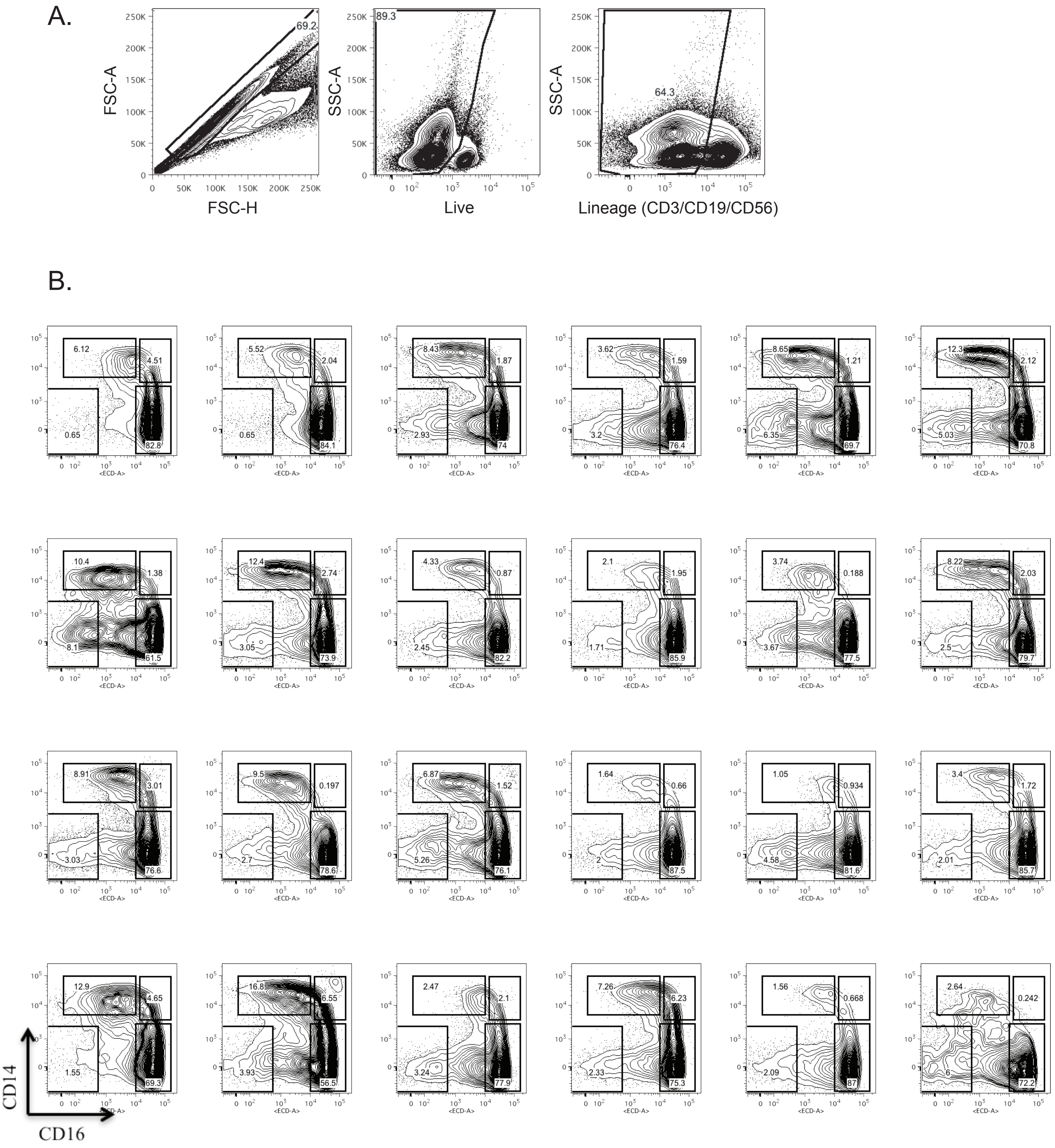

S. Figure 1. three blood myeloid subpopulations were defined by expression of HLA-DR, CD14, CD16, and CD11c: CD14+CD16- classical monocytes, CD14dimCD16+ non-classical monocytes, and CD11c+ myeloid dendritic cells (mDCs) (Flow plots from ART patients described in Table 3).
