# Supplementary material for: Levels of circulating myeloid subpopulations and of heme oxygenase-1 do not predict CD4+ T cell recovery after the initiation of antiretroviral therapy for HIV disease
Source: AIDS Res Ther. 2014 Aug 5;11:27. doi: 10.1186/1742-6405-11-27 (PMC4150425; doi:10.1186/1742-6405-11-27)
Supplement: Additional file 2: Figure S2 — Antiretroviral therapy is associated with decreased T cell activation. Column statistics were performed by 1-way ANOVA on the % expression of HLA-DR and CD38 on (A) CD4+ T cells and (B) CD8+ T cells. [file 1742-6405-11-27-S2.pdf]

Supplemental Figure 2

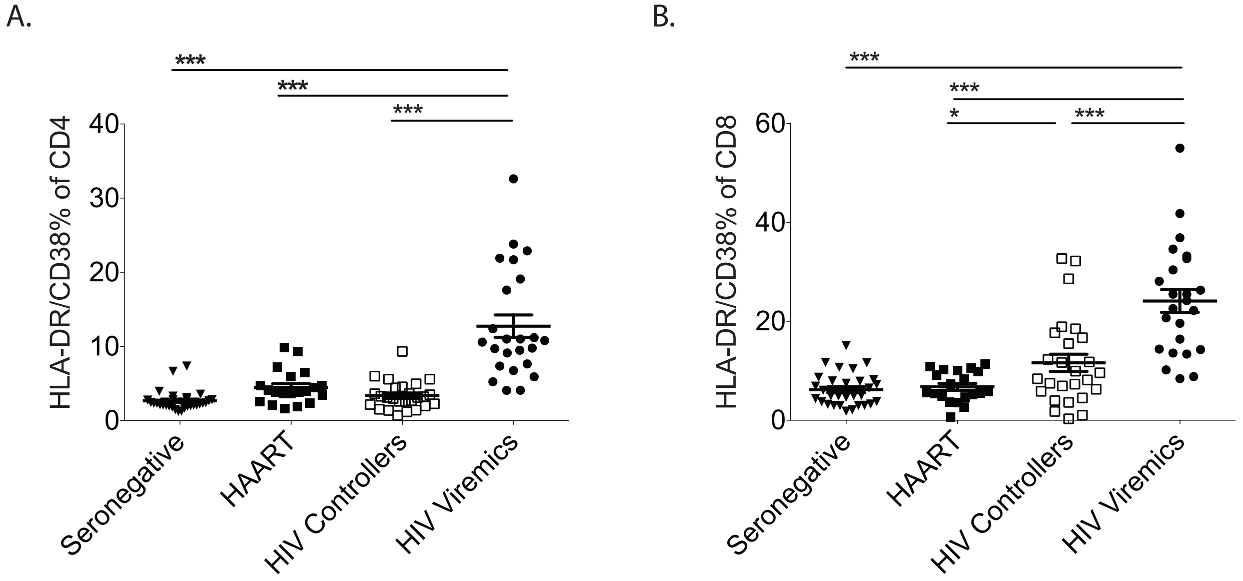

S Figure 2. Antiretroviral therapy is associated with decreased T cell activation.
Column statistics were performed by 1-way ANOVA on the % expression of HLA-DR and CD38 on (A) CD4+ T cells and (B) CD8+ T cells
