# Supplementary material for: Levels of circulating myeloid subpopulations and of heme oxygenase-1 do not predict CD4+ T cell recovery after the initiation of antiretroviral therapy for HIV disease
Source: AIDS Res Ther. 2014 Aug 5;11:27. doi: 10.1186/1742-6405-11-27 (PMC4150425; doi:10.1186/1742-6405-11-27)
Supplement: Additional file 3: Figure S3 — Monocyte populations are defined by distinct cell surface receptors. Thawed PBMC samples from Early ART Patients (ART time point 1 in Table 3) (n = 24) were analyzed for cell surface expression of monocyte markers. Plots depict staining intensity (geometric mean fluorescence intensity) of various myeloid markers (HO-1, HLA-DR, CD11b, CD11c, CD33, and CD124) in CD14hiCD16-, CD14dimCD16+, and CD14-CD16- cells (column statistics performed by 1-way ANOVA, with asterisks representing the significance to the decimal place of the p value). [file 1742-6405-11-27-S3.pdf]

Supplemental Figure 3

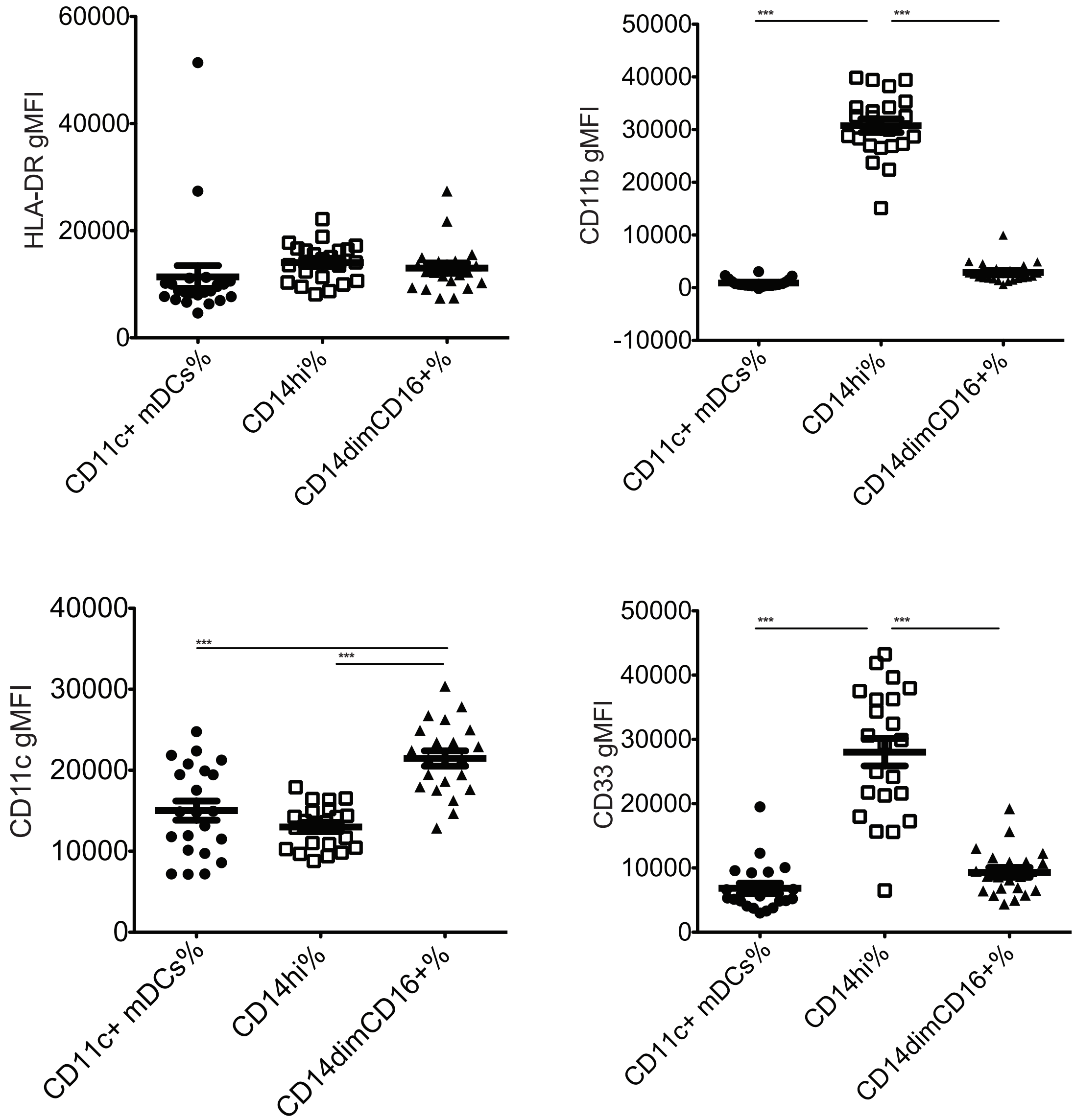

S. Figure 3. Monocyte populations are defined by distinct cell surface receptors Thawed PBMC samples from Early ART Patients (ART time point 1 in Table 3) (n= 24) were analyzed for cell surface expression of monocyte markers. Plots depict staining intensity (genometric mean fluorescence intensity) of various myeloid markers (HO-1, HLA-DR, CD11b, CD11c, CD33, and CD124) in CD14hi, CD14dimCD16+, and CD14-CD16- cells (column statistics performed by 1-way ANOVA, with asterisks representing the significance to the decimal place of the p value).
