# Supplementary material for: Levels of circulating myeloid subpopulations and of heme oxygenase-1 do not predict CD4+ T cell recovery after the initiation of antiretroviral therapy for HIV disease
Source: AIDS Res Ther. 2014 Aug 5;11:27. doi: 10.1186/1742-6405-11-27 (PMC4150425; doi:10.1186/1742-6405-11-27)
Supplement: Additional file 4: Table S1 — Antibodies used for flow cytometry staining. [file 1742-6405-11-27-S4.pdf]

Supplemental Table 1

Antibodies used for flow cytometry staining

Antigen specificity	Clone	Conjugate(s)	Vendor
CD3	SP34-2	Pacific Blue, APC	BD Biosciences
CD4	S3.5	PE/Texas Red	Invitrogen
CD8	3B5	Qdot 605	Invitrogen
CD11b	ICRF44	PECy7	BD Biosciences
CD11c	B-LY6	V450	BD Biosciences
CD14	RMO52	ECD	Beckman Coulter
CD16	3G8	Alexa 700, APC	Invitrogen
CD19	HIB19.1	Alexa 700, APC	BD Biosciences
CD33	HIM3-4	FITC	BD Biosciences
CD38	HB7	PE	BD Biosciences
CD45RA	L48	PECy7	BD Biosciences
CD56	NCAM16.2	FITC, APC	BD Biosciences
CD124	HIL4R-M57	PE	BD Biosciences
CD195 (CCR5)	2D7	PECy 5	BD Biosciences
CD197 (CCR7)	3D12	APC-eFluor 780	eBiosciences
CD279 (PD-1)	EH12.2H7	Alexa 647	BioLegend
HLA-DR	L243	FITC, APCCy7	BD Biosciences
HO-1	AB13243	Unconjugated	Abcam
anti-Rabbit IgG	Polyclonal	Qdot 605	Invitrogen
